# Knowledge and behaviours regarding the infant oral health visit among dental nurses and dental hygienists in Ireland

**DOI:** 10.1007/s40368-026-01164-4

**Published:** 2026-02-14

**Authors:** Maeve Brogan, Yodara Abdalla, Leena Al-Bayati, Lisa Blaney, Ahmed Elrayah, Nour Gharib, Nick Jadidi, Angela Jiang, Darius Sagheri, Maria Van Harten

**Affiliations:** 1 Dublin Dental University Hospital, Dublin, Ireland; 2 Trinity College Dublin, Dublin, Ireland

**Keywords:** Infant, Paediatric dentistry, Dental hygienists, Dental nurses, Health knowledge, Attitudes, Preventive dentistry, Ireland

## Abstract

**Aims:**

To explore the knowledge and behaviours regarding the infant oral health visit among dental nurses (DNs), including registered (RDNs) and non-registered (NRDNs), and dental hygienists (DHs) in Ireland.

**Methods:**

This cross-sectional, mixed-methods study assessed the knowledge and behaviours of Irish DNs and DHs regarding infant oral health visits. Quantitative survey data was analysed using IBM SPSS version 29, while qualitative focus group discussion was thematically analysed to explore barriers and practices in early oral health promotion.

**Results:**

The overall response rate was 38%, with 122 responses received from 321 questionnaires distributed. Results indicated that parental education was the most common procedure performed (22.3%) and fluoride interventions were least commonly performed. Lack of parental requests (32.5%) was identified as the primary barrier. RDNs were significantly more likely to treat paediatric patients (74.5%) than DHs (40%). Those who had received both theoretical and clinical training were significantly more likely to provide care to infants (78.9%). Public DNs and DHs treated significantly more children (89.7%) than private (44.2%). Older graduates (1960–1979) were more aware of the recommended first dental visit (83.3%).

**Conclusion:**

The present study highlights significant gaps in the knowledge and behaviours of DHs and DNs regarding infant oral health in Ireland, with inconsistencies in education, limited clinical exposure, and low parental awareness serving as key barriers. Variability in training and practice settings, particularly the greater involvement of public-sector DNs in infant care, underscores disparities in service delivery. Additionally, inconsistent fluoride varnish application and a lack of consensus on the ideal age for a first dental visit further hinder the timely delivery of early preventive infant oral healthcare. Addressing these challenges through evidence-based education for DNs and DHs will ensure equitable access to preventive care for all infants.

## Introduction

A recent study in Ireland highlighted that a child’s first dental visit by 12 months-of-age should be a key priority on the health agenda in Ireland (Duane et al. 2017). Early childhood caries (ECC) is the most prevalent chronic disease affecting young children representing a global public health concern, occurring at a rate five times higher than asthma (Duque et al. 2022; Duane et al. 2017). Previous research has demonstrated that dental caries prevalence increased from 8% at 18 months to 23% at 36 months of age, underscoring the need for early intervention (Gussy et al. 2016).

Prevention of ECC is therefore critical, and dental hygienists (DHs) and dental nurses (DNs) in Ireland play a key role in shaping early oral health practices. According to Irish Dental Council Guidelines, the role of the DH involves providing guidance on oral health, organising oral health promotion programs, offering smoking cessation advice, providing plaque control strategies and conducting dietary analysis to prevent dental caries (Dental Council of Ireland 2022). Similarly, DNs are involved in providing patients with relevant health promotion guidance, empowering individuals to take greater control of their health and, in turn, improve their overall well-being (World Health Organization 2025).

The guideline of the first dental visit before age 1 year or when the child’s first tooth erupts has been adopted by numerous professional bodies (AAPD 2024; Bhaskar et al. 2014; FDI 2020). The rationale for the infant oral health visit is manifold: early prevention, parental education, establishing a dental home, and reducing future treatment needs (Weber-Gasparoni 2019). However, among dental professionals, there is a clear discrepancy in when they believe the timing of the first visit should be. A recent Irish study (Djokic et al. 2018) found that only 58% of non-paediatric dentists were aware of the guideline, echoing similar findings reported in Canada (Canadian Dental Association 2025).

It is evident that health education should begin at an early age to track a child’s development and prevent potential health issues (Saccomanno et al. 2023). However, studies have identified knowledge gaps in dental practitioners’ understanding and prevention of dental caries in infants, underscoring the need for improvement (Alrowaili 2021). Moreover, discrepancies between knowledge and behaviour persist, with studies demonstrating differences in dentists’ perceived effectiveness of preventive methods, such as fluoride and sealants, compared to their actual practices (Lin et al. 2010). These studies, despite relying on self-reported data, underscore the global need for targeted training among dental professionals to close knowledge gaps and and ensure the delivery of consistent, effective preventive care for infant oral health.  

In relation to DHs, the literature reveals clear variations in knowledge and behaviours. One study found widespread confidence in conducting caries risk assessments yet identified limited use of standardised tools and significant knowledge gaps (Francisco et al. 2013). This need to address the lack of knowledge on infant oral health among DHs is similarly underscored in various other studies (Manski and Parker 2010; Ruiz et al. 2014).

While extensive research has examined dentists’ knowledge and behaviours regarding infant oral health, little attention has been given to DHs, and notably, no studies have assessed DNs’ knowledge or practices. With the growing emphasis on preventive dentistry (Garcia and Sohn 2012), the role of DHs and DNs in educating parents and caregivers is becoming increasingly important.

The present study was the first of its kind in Ireland to examine both DNs and DHs, addressing a significant gap in the literature. Existing literature often overlooks the critical role these health professionals play in patient education, guidance, and early intervention. The study aimed to assess DHs’ and DNs’ knowledge and behaviours regarding infant oral health, identify barriers to care, and explore how they perceive and implement best practices. Additionally, it investigated how training methods, years of experience, and practice settings influenced the incorporation of infant oral health visits into routine care. Data was collected via an online survey of DHs and DNs in Ireland, followed by a focus group discussion.

By evaluating current knowledge and practices, the study seeks to enhance education, standardise preventive strategies, and ultimately contribute to improved early childhood oral health outcomes in Ireland.

## Methods

The present study, approved by the Trinity College Dublin Research Ethics Committee on 29th January 2025, employed a cross-sectional, mixed-methods design to assess the knowledge and practices of DHs and DNs in Ireland regarding infant oral health visits. A mixed-methods approach was chosen to provide a more comprehensive understanding by combining quantitative survey data with qualitative insights from focus groups. A convenience sampling method was used, targeting DNs and DHs registered with the Irish Dental Nurses Association (IDNA) and the Irish Dental Hygienists Association (IDHA), along with oral healthcare workers in dental practices across Ireland.

### Questionnaire

A 12-item questionnaire developed using a survey instrument based on validated tools (Djokic et al. 2018) was employed to gather quantitative data on participants’ knowledge, attitudes, and practices related to infant oral health visits. This questionnaire was adapted for DHs and DNs. The survey was conducted over four weeks using Qualtrics and consisted of multiple-choice and open-ended questions. It was distributed via email to IDNA and IDHA members, as well as to dental practices across Ireland using contact information from the 2025 Dental Council of Ireland Register, , with follow-up telephone calls to encourage participation. Participant selection included participants working in dental clinics in Ireland, with at least one year of experience (Table 1). Two gatekeepers were involved—the President of the IDNA and the Employment and Website Officer for the IDHA. Participants were provided with detailed information about the study’s objectives, and written informed consent was obtained for both stages of the study. Participants were provided with a Qualtrics web link and were assured of anonymity and confidentiality, with the option to withdraw at any stage. The data was stored for a month after survey completion in compliance with GDPR (General Data Protection Regulation). Incomplete or unsubmitted responses were excluded from analysis.

**Table 1:** Inclusion and exclusion criteria

Inclusion criteria	Exclusion criteria
Profession: Participants must be qualified or currently practicing dental hygienists or dental nurses	Non-Oral Health Professionals: Any individual who is not a dental hygienist or dental nurse (e.g. administrative staff)
Location: Participants must be working in dental practices, clinics, or healthcare settings in Ireland	Non-Ireland Based: Oral healthcare workers not currently practicing in Ireland
Experience: Must have at least one year of professional experience in oral healthcare	Students or Trainees: Participants who are still in training

### Focus group

Involvement in a follow-up focus group was offered to questionnaire participants, with an opt-in question at the end. Seven participants attended a 45-minute Zoom session held on a later date. A pre-prepared interview guide directed the discussion to explore questionnaire trends in depth. Participants were assigned pseudonyms to ensure anonymity and were informed that they could withdraw at any time. All data were securely stored on password-protected devices, to which only authorised research team members had access.

### Data analysis

Quantitative survey data were entered into Microsoft Excel (Microsoft Corporation, Redmond, WA, USA) and analysed in IBM SPSS Statistics version 29 (IBM Corp., Armonk, NY, USA) to compute descriptive statistics. The Chi-squared test was used due to the categorical nature of the variables in this study (*p* < 0.05 was considered significant). Qualitative focus group data were transcribed from the audio recording and analysed thematically.

## Results

### Questionnaire results

The overall response rate was 38%, with 122 responses received from 321 questionnaires distributed. The demographic breakdown was: 30.7% DHs (*n* = 35), 44.7% RDs (*n* = 51), and 24.6% NRDNs (*n* = 28). Eight responses were excluded due to incomplete surveys. Questionnaire responses are summarised in Table 2.

**Table 2:** Summary of questionnaire responses from dental hygienists (DH), dental nurses (DN) and non-registered dental nurses (NRDN)

Question	Response option	DH	DN	NR DN	Total
Q1 What is your role as an oral healthcare worker?	Dental hygienist	35 (30.7%)			114
	Registered dental nurse		51 (44.7%)		
	Non-registered dental nurse			28 (24.6%)	
Q2 Year of graduation from undergraduate course:	1960–1979	4	2	0	6 (5.3%)
	1980–1989	1	3	0	4 (3.5%)
	1990–1999	4	1	2	7 (6.1%)
	2000–2009	7	6	3	16 (14%)
	2010–2019	10	16	4	30 (26.3%)
	2020–2024	9	21	5	35 (30.7%)
	No undergraduate degree	0	0	16	16 (14%)
Q3 How long have you been practicing in Ireland (years)?	1–5	9	18	19	46 (40.4%)
	6–10	7	14	3	24 (21.1%)
	11–15	5	5	1	11 (9.6%)
	16–20	5	6	3	14 (12.3%)
	21+	9	8	2	19 (16.7%)
Q4 Where did you receive your post registration degree from?	Ireland	30	46	23	103 (90%)
	Outside Ireland	5	4	2	11 (9.6%)
Q5 Number of hours per week in clinical practice:	0–10	2	2	3	7 (6.1%)
	11–20	5	3	5	13 (11.4%)
	21–30	21	5	3	29 (25.4%)
	31+	7	41	17	65 (57%)
Q6 What is your clinical practice setting?	Private practice	24	28	25	77 (67.5%)
	Public setting (community/hospital)	8	19	2	29 (25.4%)
	Both	3	3	1	7 (6.1%)
	Other	0	1	0	1 (0.9%)
Q7 Regarding the infant oral health visit, what training did you receive during your undergraduate dental education?	Theory	24	19	7	50 (43.9%)
	Clinical Practice	0	1	2	3 (2.6%)
	Both	8	24	6	39 (34.2%)
	None	3	7	12	22 (19.3%)
Q8 Do you see (examine and/or treat) children aged 0–36 months in your clinical practice?	Yes	14	38	15	67 (58.8%)
	No	21	13	13	47 (41.2%)
Q9 How often do you see children aged 0–36 months in your clinical practice?	1 child / week	2	13	6	21 (18.4%)
	1 child / month	4	13	9	26 (22.8%)
	1 child every 6 months	7	9	6	22 (19.3%)
	1 child / year	19	15	5	39 (34.2%)
	Don't see children this young in my practice	0	4	2	6 (5.3%)
Q10 Which of the following do you routinely carry out as part of a visit for a child aged 0–36 months? (select all that apply)	Examine for dental caries	7	30	14	51 (13.9%)
	Examine for normal dental development	8	33	16	57 (15.5%)
	Give dietary advice	26	27	14	67 (18.2%)
	Educate parents about caries and prevention	25	36	21	82 (22.3%)
	Give oral hygiene instructions	19	29	12	60 (16.3%)
	Evaluate fluoride needs	4	14	8	26 (7.1%)
	Place fluoride varnish	1	11	4	16 (4.3%)
	Other (please specify)	4	3	2	9 (2.4%)
	None of the above	0	0	0	0
Q11 What is your reason for not seeing children aged 0–36 months?	Parents do not request appointments for children this young	28	28	9	65 (32.5%)
	Prefer to refer infants to paediatric dentist	4	10	4	18 (9%)
	Parents do not see the value of the first dental visit at 12 months of age	17	18	3	38 (19%)
	It is another healthcare provider's responsibility to perform infant oral health visits	4	1	0	5 (2.5%)
	Not sure what to do at a visit for 12-month-old or younger	5	4	1	10 (5%)
	It is not cost-effective seeing infants	5	7	2	14 (7%)
	Most children do not need dental care at age 1	1	4	3	8 (4%)
	Do not enjoy seeing infants	2	3	2	7 (3.5%)
	My dental practice is too busy	0	4	2	6 (3%)
	It is too time consuming seeing infants	1	4	3	8 (4%)
	Not comfortable seeing infants	4	1	1	6 (3%)
	Other (please specify)	3	5	7	15 (6.5%)
Q12 At what age do you believe children should have their first dental visit?	0–12 months	9	16	7	32 (28.1%)
	Age 1	9	14	4	27 (23.7%)
	Age 2	13	9	5	27 (23.7%)
	Age 3	2	8	4	14 (12.3%)
	Age 4	1	4	5	10 (8.8%)
	Age 5	0	0	2	2 (1.7%)
	Age 6	0	0	2	2 (1.7%)

Regarding clinical practice settings, 67.5% of participants (*n* = 77) worked in private practice, 25.4% in public settings (*n* = 29), 6.1% in both (*n* = 7), and 0.9% in other roles (*n* = 1).

Participants graduated between 1960 and 2024, with 30.7% completing their degrees between 2020–2024. Additionally, 14% had no undergraduate training. Most respondents (*n* = 103) obtained their post-registration degree in Ireland, while 9.6% had trained abroad.

 Regarding workload, 57% worked full-time (≥ 31 hours per week), 25.4% worked 21–30 hours, 11.4% worked 11–20 hours, and 6.1% worked 0–10 hours. Training exhibited substantial variability, with 43.9% receiving only theoretical instruction, 34.2% receiving both clinical and theoretical training, 2.6% obtaining clinical training only, and 19.3% having no formal training.

The most cited barrier was lack of parental requests (32.5%), followed by perceived lack of necessity (19%) and preference for specialist referrals (9%). Less commonly cited factors included role delegation, uncertainty about interventions, financial concerns, belief that young children don’t require care, busy practice, lack of enthusiasm, excessive time commitment and personal discomfort. Additionally, 6.5% responded in relation to ‘Other’, with responses including working in specialised or A&E settings (Table 2).

The most frequent service provided by DHs and DNs was parental education (22.3%), followed by dietary advice (18.2%). Others included oral hygiene instructions (16.3%), assessment of normal dental development (15*.5*%), and caries examination (13.9%). Fluoride evaluation (7.1%) and varnish application (4.3%) were the least frequently performed. Additionally, 2.4% selected ‘other,’ which included emergency child exams, tongue-tie release, and acclimatising children to the dental environment.

A statistically significant association was present between professional designation and seeing children aged 0–36 months (*P* = 0.0495). RDNs were significantly more likely to see paediatric patients (74.5%,), whereas DHs were significantly less likely (40%) (Table 3).

**Table 3:** Examination and treatment of infants (0–36 months) by dental nurses and dental hygienists

Do you see (examine and/or treat) children aged 0–36 months in your clinical practice?	Non-registered dental nurse (%)	Registered dental nurse (%)	Dental hygienist (%)	Chi-squared test value	*P* value
Yes	53.6	74.5	40.0	10.62	0.00495(significant at *P* < 0.05)
No	46.4	25.5	60.0		

**Fig. 1:**
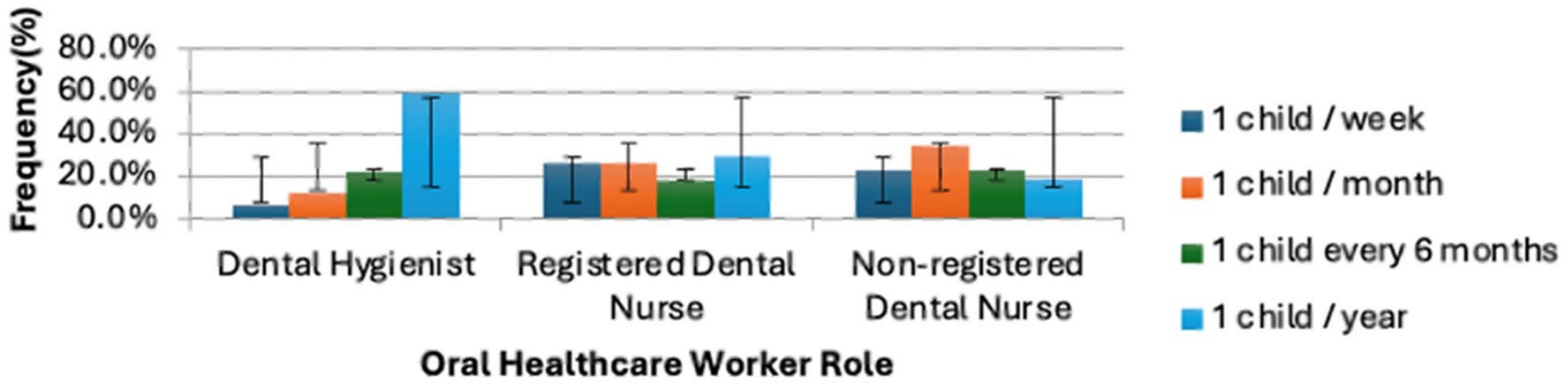
Relationship between ‘Oral healthcare worker role’ and ‘Frequency of seeing children aged 0–36 months’

There was a statistically significant association between the role of an oral healthcare worker and the likelihood of seeing infants (*P* = 0.022). DHs were the least likely to see children frequently, with 6.3% seeing at least 1 child per week. RDNs and NRDNs were similar in their frequency of seeing children, with 26% and 23.1% respectively seeing one child per week (Fig. 1).

**Fig. 2:**
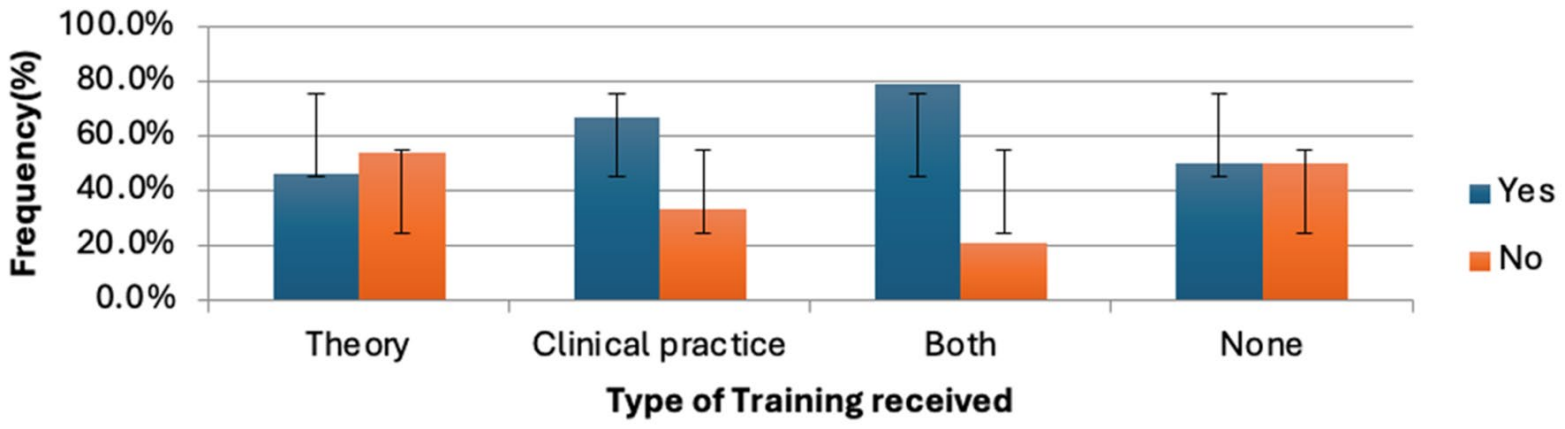
Relationship between ‘Type of training received’ and ‘Treatment of children aged 0–36 months’

A statistically significant association was present between undergraduate training type and treating children aged 0–36 months (*P* = 0.015). Respondents with both theoretical and clinical training were significantly more likely to treat infants (78.9%) compared to those with theory-only training (46%), who were significantly less likely to treat paediatric patients (Fig. 2).

**Fig. 3:**
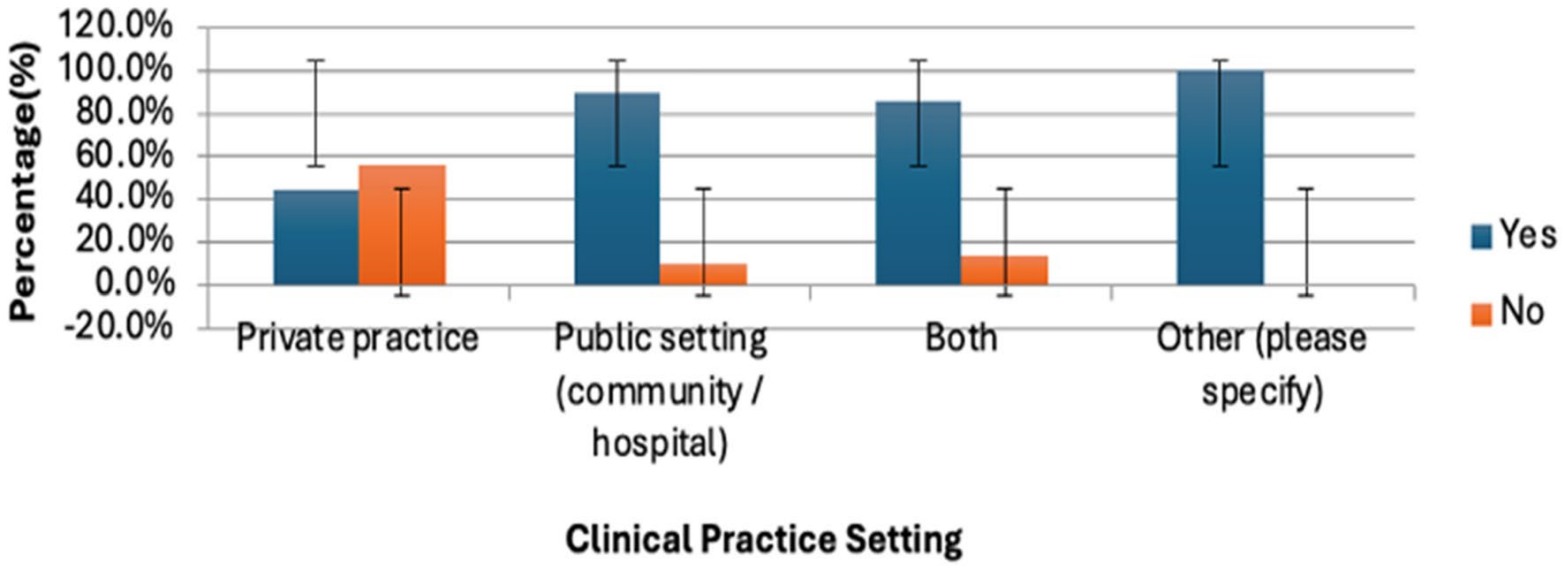
Relationship between ‘Clinical practice setting’ and ‘Seeing children aged 0–36 months’

A statistically significant association was present between practice setting and treating paediatric patients (*P* = 0.015). In private practice, 55.8% did not treat children, while 44.2% did. In contrast, a significant proportion of public practice respondents (89.7%) treated paediatric patients, with only 10.3% not providing care (Fig. 3).

**Fig. 4:**
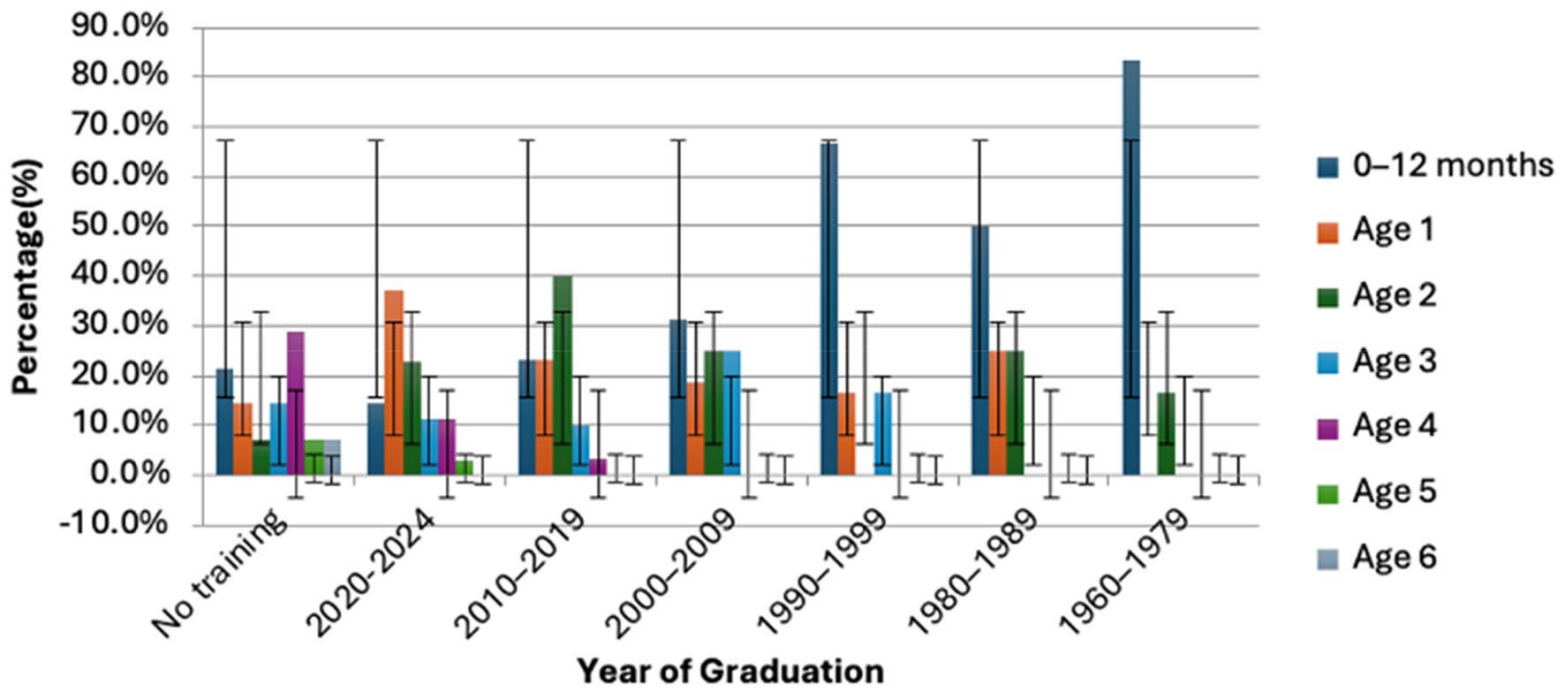
Relationship between ‘Year of graduation’ and ‘Belief on what age children should have their first dental visit’

Graduation year influenced knowledge of the recommended first dental visit. Earlier graduates were more likely to endorse a 0–12-month visit, with 83.3% of those from 1960–1979 doing so, compared to 14.3% of 2020–2024 graduates (Fig. 4).

### Focus group results

The focus group discussion topics were thematically organised to address training and educational gaps, clinical exposure and the frequency of infant visits, routine practices in infant oral health visits, barriers to providing infant oral healthcare, beliefs regarding the ideal age for the first dental visit, and recommendations.

Various responses within each theme were obtained.

1. Training and educational gaps

Responses regarding the adequacy of training received indicated considerable variability in both training and experience.

“ We don’t get enough training on infant oral health. More practical experience would really help us feel more confident.”

“ My training adequately prepared me for my job. However, I work in a hospital and was trained in the hospital so I feel like it trained me for the job I have. Other colleagues of mine work in private practice and would disagree with this.”

2. Clinical exposure and frequency of infant visits

Participants noted that increased frequency increased their confidence in seeing infants.

“The more you see infants, the more prepared you feel to talk to parents about oral health matters.”

In relation to the relevance of clinical practice setting, participants were not surprised that public practices saw more infants than private settings.

“In public practices, we see way more young kids. It’s really just because it’s more affordable for families. In private practice, if a child needs sedation or general anaesthesia, it adds to the overall cost.”

3. Routine practices in infant oral health visits

Many participants emphasised the importance of education.

“Education and advice are key components of infant oral health. For example, a hygienist visited my son’s school and showed them how to brush their teeth. Afterwards, many parents started bringing their children to the dentist. It just goes to show how important education is.”

4. Barriers to providing infant oral healthcare

Many barriers were noted by participants ranging from embarrassment to financial reasons.

“There’s an element of embarrassment. Parents feel ashamed if their child’s teeth are in poor condition.”

“Parents don’t care. They think deciduous teeth don’t matter since they’ll be replaced anyway.”

“Parents wait until the HSE sees their children in primary school. Unless there’s an emergency, they don’t bring them in earlier. Many private dentists defer the provision of infant oral health care to public services.”

5. Beliefs about the ideal age of the first dental visit

In relation to the guideline on the first dental visit, some participants noted discrepancy in guidelines.

“There’s conflicting information in the literature regarding the recommended age of the first dental visit”

When asked why they believed those who graduated years ago had more experience, participants attributed it largely to continuing professional development (CPD).

“Those who graduated years ago have so much more experience. A big factor is that they have done a lot of CPD training. They really know everything – I’m in awe of them.”

6. Recommendations

Participants provided several recommendations to improve infant oral health education and practice:

“I’d be interested in CPD training, but it would need to be funded by the practice. That’s why hospital-based DNs tend to have more CPD than those in private practice – it’s covered for them.”

Other suggestions included increased clinical exposure to infants:

“Clinical exposure to infants during undergraduate training would be extremely beneficial.”

## Discussion

### Training and educational gaps in infant oral health

The variation in responses amongst participants with theoretical training, both theoretical and clinical training, or no training at all highlights inconsistencies in educational preparation. DHs were more likely to receive theory-only training, while RDNs had more comprehensive clinical and theoretical training. Notably, those trained in both modalities were significantly more likely to treat infants than those with theory-only training.

The discrepancy in perceived job preparedness underscores the need to address gaps in formal education.The findings of the present study are consistent with prior research demonstrating that education enhances professional confidence. For example, in a cross-sectional study, Calhoun et al. (2023) reported that higher levels of education predicted a greater likelihood of DHs providing anticipatory guidance to parents. 

### Clinical exposure and frequency of infant visits

The present study found significant differences in clinical exposure, with RDNs seeing more infant patients than DHs. Infant visit frequency also varied, with few DHs seeing one child per week. Those working more than 31 hours per week were more likely to treat children than those working only 10 hours per week*.* Frequent exposure correlated with increased confidence in educating parents.

Practice setting also influenced patient demographics. DHs and DNs in public practice treated more infants than those in private practice, aligning with research showing public services handle most paediatric dental care due to limited private practice options (Auld 2023). The disparity between public and private practice highlights the need for standardised formal teaching on infant oral health.

### Routine practices in infant oral health visits

The present study underscores the complementary roles of DHs and DNs in infant oral health, particularly in parent education and preventive care to support the dentist’s role. As poor oral hygiene is a significant risk factor for ECC (Kirthiga et al. 2019), parental education on prevention is critical. However, fluoride varnish application remains underutilised, despite being a critical preventive measure, consistent with previous studies reporting inconsistent fluoride varnish application rates (Djokic et al. 2018; Manski and Parker 2010).

The present study also revealed significant variations in knowledge and consistency related to routine dental practices, aligning with previous research which examined the consistency of caries prevention recommendations and found that while two-thirds of professionals prioritised oral hygiene education, only 18% emphasised dietary advice (Løken et al. 2016).

### Barriers to providing infant oral healthcare

The primary barrier to infant oral healthcare identified was the lack of parental initiative in requesting appointments, often due to a lack of awareness, concern, or even embarrassment. This aligns with a recent Irish study in which 41% of non-paediatric dentists identified parental unawareness as a major obstacle (Djokic et al. 2018). Most parents rely on HSE (Health Service Executive) referrals unless there is an emergency,  consistent with a systematic review reporting that infants are usually seen only for treatment rather than for preventive care (Bhaskar et al. 2014).

 This finding is further supported by a recent Irish study (Duane et al. 2017),which reported that while the HSE provides dental services up to age 16 years, non-emergency cases often experience delays. Routine school referrals typically occur between ages 6 and 8 years, so often infants are not seen until then. Other less frequently cited barriers; such as financial constraints also align with previous research, as well as uncertainty about what to expect or do at a 12 month dental visit (Schroth et al. 2015; Ruiz et al. 2014).

### Beliefs about the ideal age for a first dental visit

The variation in recommended ages for a child’s first dental visit highlights inconsistencies in professional knowledge and adherence to guidelines, emphasising the need for clearer recommendations. The present study found significant differences in beliefs about the ideal timing. International research indicates that while DHs often prioritise topics like diet and pacifier use, fewer (48%) emphasise the importance of a 12-month visit (Calhoun et al. 2023).

Findings from the present study indicate this discrepancy may be influenced by experience levels. For example, graduation year was linked to knowledge, with those graduating between 1990 and 1999 more likely to recommend early visits, likely reflecting their extensive clinical expertise. This aligns with studies showing that greater experience correlates with higher knowledge scores (Alrowaili 2021; Manski and Parker 2010). Respondents also noted inconsistencies in professional guidelines, highlighting the need for improved quality guidelines (Verdugo-Paiva et al. 2024). This is reinforced in the literature with online sources not always aligning with professional advice (Bhaskar et al. 2014).

### Recommendations

The present study highlights the need to enhance the knowledge of DHs and DNs in the context of educational research, with findings suggesting that additional education through dental curricula and continuing education would be well received, consistent with prior research which reported that approximately 90% of DHs would be interested in CPD (Manski and Parker 2010; Clovis et al. 2012). Another recommendation was for non-registered dental nurses (NRDNs) to complete a short foundational course before starting work as a DN to ensure they acquire essential knowledge.

Furthermore, incorporating practical training in infant oral health into undergraduate DH and DN programmes could better prepare future professionals. This importance of obtaining both theory and clinical training is highlighted in the literature (Ruiz et al. 2014).

### Limitations

The present study has several limitations, primarily stemming from biases associated with self-reported data, including self-selection and recall bias, which may affect the reliability of findings. Response bias may have occurred due to non-participation, potentially leading to an overrepresentation of certain perspectives. Additionally, the use of convenience sampling limits the generalisability of results. Time constraints further posed a challenge to data collection due to the delay in the ethics form approval.

.The web-based survey also encountered several limitations, including reluctance from DH associations to distribute the link, technical difficulties, and respondents’ lack of necessary technical skills.

Furthermore, there was a lack of clarity associated with some survey questions. Question 7 on undergraduate training may have led to overreporting, as NRDNs likely had no formal training. Participants may have overreported treatments they provide, particularly procedures like tongue-tie releases and emergency exams, which fall outside the scope of practice for DNs and DHs as per Irish Dental Council guidelines.

Ambiguities in terminology, such as the definition of “children” (0–36 months) and distinctions between age categories (e.g., 0–12 months vs. 1 year), also contributed to confusion. Additionally, multiple-choice questions (Q10 and Q11) made it challenging to categorise responses accurately.

## Conclusion

This study highlights critical gaps in the knowledge and behaviours of DHs and DNs regarding infant oral health visits in Ireland. The willingness of these professionals to see paediatric patients was significantly influenced by their practice setting, training modality, and professional role. Notably, those who received both theoretical and clinical training exhibited greater confidence and engagement, underscoring the importance of a structured curriculum that includes hands-on practical experience.

Findings also revealed that public-sector DNs were more engaged with young patients than their private-sector counterparts, emphasising the need for standardised training across all settings. Barriers such as low parental awareness further hinder early dental visits, reinforcing the necessity of targeted public education and improved dissemination of clinical guidelines. Despite efforts in parental education, preventive measures like fluoride varnish remain underutilised, highlighting inconsistencies in guideline adherence.

The absence of consensus on the recommended age for a first dental visit further underscores the need for clearer, standardised guidelines. Expanding access and funding for CPD courses would enhance professional development and strengthen infant oral health services. Addressing knowledge gaps and increasing clinical exposure for DHs and DNs would likely contribute to a paradigm shift in infant oral healthcare, ultimately improving long-term outcomes. Standardised, evidence-based interventions are essential to ensure timely and equitable care for all infants, reaffirming the crucial role of oral health professionals in early prevention.

## Data Availability

No datasets were generated or analysed during the current study.
